# Mitochondrial Energy Metabolism in the Regulation of Thermogenic Brown Fats and Human Metabolic Diseases

**DOI:** 10.3390/ijms24021352

**Published:** 2023-01-10

**Authors:** Yukimasa Takeda, Yoshinori Harada, Toshikazu Yoshikawa, Ping Dai

**Affiliations:** 1 Department of Cellular Regenerative Medicine, Graduate School of Medical Science, Kyoto Prefectural University of Medicine, 465 Kajii-cho, Kawaramachi-Hirokoji, Kamigyo-ku, Kyoto 602-8566, Japan; 2 Department of Pathology and Cell Regulation, Graduate School of Medical Science, Kyoto Prefectural University of Medicine, 465 Kajii-cho, Kawaramachi-Hirokoji, Kamigyo-ku, Kyoto 602-8566, Japan; 3 Louis Pasteur Center for Medical Research, 103-5 Tanaka-Monzen-cho, Sakyo-ku, Kyoto 606-8225, Japan

**Keywords:** brown adipose tissue, brown adipocyte, beige adipocyte, adipocyte browning, uncoupling protein 1, mitochondria energy metabolism, metal ion, obesity, metabolic diseases

## Abstract

Brown fats specialize in thermogenesis by increasing the utilization of circulating blood glucose and fatty acids. Emerging evidence suggests that brown adipose tissue (BAT) prevents the incidence of obesity-associated metabolic diseases and several types of cancers in humans. Mitochondrial energy metabolism in brown/beige adipocytes regulates both uncoupling protein 1 (UCP1)-dependent and -independent thermogenesis for cold adaptation and the utilization of excess nutrients and energy. Many studies on the quantification of human BAT indicate that mass and activity are inversely correlated with the body mass index (BMI) and visceral adiposity. Repression is caused by obesity-associated positive and negative factors that control adipocyte browning, de novo adipogenesis, mitochondrial energy metabolism, UCP1 expression and activity, and noradrenergic response. Systemic and local factors whose levels vary between lean and obese conditions include growth factors, inflammatory cytokines, neurotransmitters, and metal ions such as selenium and iron. Modulation of obesity-associated repression in human brown fats is a promising strategy to counteract obesity and related metabolic diseases through the activation of thermogenic capacity. In this review, we highlight recent advances in mitochondrial metabolism, thermogenic regulation of brown fats, and human metabolic diseases.

## 1. Introduction

The impact of obesity on morbidity, mortality, and medical costs is enormous [1]. The prevalence of Western diets and overnutrition has resulted in a serious epidemic of obesity and metabolic diseases including type 2 diabetes, cardiovascular diseases, and stroke. Obesity is also associated with the risk of developing numerous cancers, which utilize glucose as the main energy source for growth [2,3]. Obesity-associated metabolic syndrome is characterized by three or more of the following conditions: overweight, hyperglycemia, hypertriglyceridemia, hypertension, and low levels of high-density lipoprotein (HDL) cholesterol [4]. Apart from lifestyle modifications, calorie restriction, and physical exercise, no other fundamental option exists to treat obesity and obesity-associated metabolic syndrome if these interventions are not sufficiently implemented. Bariatric surgery is substantially effective for the long-term prevention of obesity; however, most people who are overweight may harbor hesitation as they might perceive it as a major surgery [5]. Anti-obesity medications are an attractive alternative for reducing body weight; however, long-term use raises concerns about safety and efficacy [6,7]. Thus, in addition to lifestyle modifications, a novel therapeutic strategy is needed for the prevention of obesity and obesity-associated metabolic diseases without adverse effects.

Adipose tissue controls many aspects of whole-body physiology including energy homeostasis, insulin sensitivity, inflammation, and maintenance of body temperature [8]. Adipose tissue is a highly plastic organ that undergoes structural, metabolic, and phenotypic switching in response to physiological cues [9]. White adipose tissue (WAT) consists of triglyceride-storing adipocytes as a primary energy source, which is advantageous for survival without a food supply for an extended period. Beyond its critical role in energy storage, WAT is an essential endocrine organ that produces various adipokines, which regulate systemic inflammation and insulin activity [10]. Brown adipose tissue (BAT) is another type of adipose tissue that consists of brown adipocytes, which participate in heat production for regulating body temperature [11]. One of the most characteristic features of brown adipocytes is that they possess enriched and metabolically active mitochondria [12]. Mitochondria are essential intracellular organelles that convert chemical energy derived from glucose and fatty acids into adenosine triphosphate (ATP) through the tricarboxylic acid (TCA) cycle, fatty acid β-oxidation, and electron transport chain (ETC). Brown adipocyte-specific expression of uncoupling protein 1 (UCP1) enables the dissipation of chemical energy in the form of heat to resist hypothermia. Mitochondrial energy metabolism in thermogenic brown fats substantially regulates heat production and consumption of circulating blood glucose and fatty acids, which are associated with whole-body energy homeostasis. Therefore, mitochondrial energy metabolism has become an attractive target for preventing obesity and related metabolic diseases, such as type 2 diabetes and cardiovascular diseases. Here, we review recent advances in mitochondrial metabolism, thermogenic regulation of brown fats, and human metabolic diseases.

## 2. Physiological Role of Brown Fats

### 2.1. Mitochondria in White and Brown Adipose Tissues

**Figure 1:**
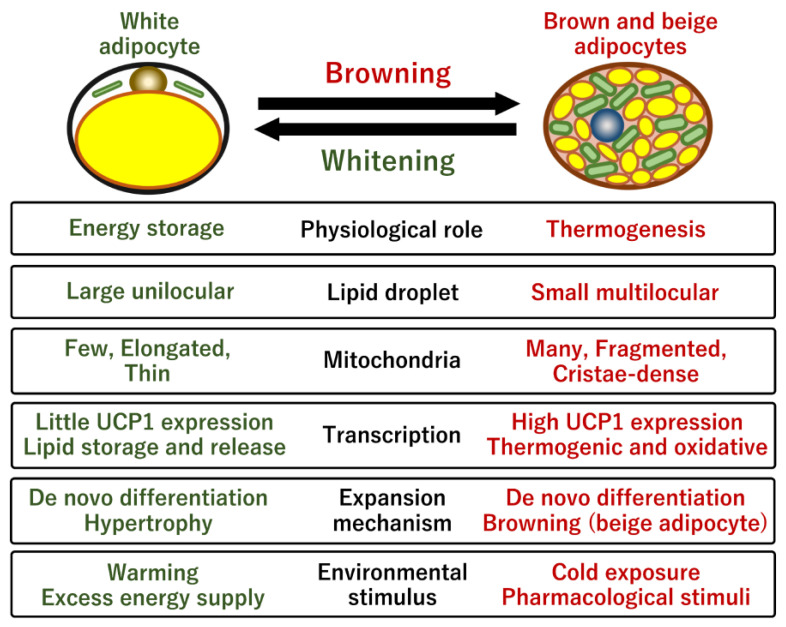
Comparison of characteristic features of white and brown adipocytes. The browning process promotes morphological change in lipid droplets, mitochondrial biogenesis, and transcriptional program for both mitochondrial oxidative respiration and thermogenesis. One of the most characteristic differences is that brown adipocytes possess enriched cristae-dense mitochondria containing a high level of iron, whereas white adipocytes possess relatively few elongated mitochond ria. Without a continuous cold stimulus, brown/beige adipocytes are phenotypically reversed into white-like adipocytes through a process termed whitening. Brown/beige adipogenesis occurs through both de novo differentiation of specific progenitor cells and transdifferentiation of dormant white adipocytes in response to either cold exposure or pharmacological stimuli. The manipulation of these two types of adipocytes is a promising approach to controlling obesity and systemic energy homeostasis.

The two types of adipocytes possess different physiological roles in terms of mitochondrial function and energy homeostasis [9]. White adipocytes are spherical cells of variable size with a unilocular droplet that stores lipids as an energy source during starvation and fasting. White adipocytes have a small number of small, elongated, and thin mitochondria that provide abundant ATP, similar to that in other tissues [13] (Figure 1). Mitochondrial dysfunctions in white adipocytes are related to adipocyte differentiation, lipogenesis, and lipolysis [14,15,16]. Emerging evidence suggests that mitochondria in white adipocytes coordinate energetic synchronization through cell-to-cell communication by intercellular mitochondrial transfer and extracellular vesicle (EV) production in WAT [17,18]. In addition, the mitochondria also modulate interorgan energy communications by releasing non-esterified fatty acids and EVs into the heart, pancreas, and liver. Thus, mitochondria of the WAT function as important mediators of intercellular and interorgan crosstalk, and their therapeutic potential is being continually studied.

In contrast, brown adipocytes possess small multilocular lipid droplets, which enable a higher lipolysis rate [19]. The mitochondria of brown adipocytes are enriched, fragmented, and round [20]. The mitochondria are larger than that in the white adipocytes, cristae-dense, and brownish due to the presence of iron-containing heme cofactor in the cytochrome oxidase, indicating their high potential for oxidative energy generation [21]. The characteristic mitochondrial morphology likely determines the efficiency of catabolic pathways, such as glycolysis, fatty acid oxidation, TCA cycle, and ETC, which are harnessed for thermogenic capacity [22]. Enhanced oxidative phosphorylation via catabolic pathways forms an effective proton gradient between the matrix and intermembrane space for ATP synthesis. However, the proton gradient is not coupled to ATP synthesis, and the process of proton leakage is a significant part of the resting metabolic rate, which suggests that mitochondrial uncoupling proteins are potential targets for obesity [23]. The inner mitochondrial membrane contains UCP1, which is specifically expressed in brown adipocytes. Inducible UCP1 dissipates the proton gradient by transferring protons across the inner membrane to produce heat. This mitochondrial uncoupling respiration is required for thermogenesis under cold conditions and for excessive energy disposal under overnutritional conditions [24]. Whole-body UCP1-deficient mice are sensitive to cold exposure but do not develop severe obesity [25]. However, gradual cold adaptation in UCP1-deficient mice suggests UCP1-independent thermogenic mechanisms [26]. The loss of other uncoupling protein homologs, UCP2 and UCP3, did not contribute to cold-induced thermogenesis, suggesting that UCP1 dependent-thermogenesis plays a significant role in brown adipocytes [27,28]. Besides the thermogenic function, the mitochondria in brown adipocytes may be involved in multiple physiological processes, such as reactive oxygen species (ROS) production, apoptosis, autophagy, inflammation, and aging [29].

### 2.2. Human BAT and Its Physiological Roles

In mammals, BAT plays a predominant role in non-shivering thermogenesis to prevent hypothermia under cold conditions without muscle shivering [24]. Thermogenic adipocytes are further categorized into brown and beige adipocytes owing to their developmental differences. Adipose tissue plasticity can be observed during cold exposure when the sympathetic nervous system (SNS) is subsequently activated to induce the development of mitochondria-enriched brown-like adipocytes within specific adipose depots; this process is termed adipocyte browning [30]. The rapid recruitment of beige adipocytes is accompanied by sympathetic nerve fiber innervation and angiogenesis to control their thermogenic capacity [31]. The browning process is highly reversible, and beige adipocytes are smoothly whitened and become dormant after cold acclimation [32,33]. In turn, the whitened adipocytes are converted into thermogenic beige adipocytes again after subsequent exposure to cold. Classical brown and beige adipocytes share morphological features, such as multilocular lipid droplets and enriched mitochondria, although UCP1 expression and UCP1-mediated thermogenic activity are more inducible and reversible in beige adipocytes (Figure 1). Classical brown adipocytes have distinct cellular origins and differentiate from myogenic factor 5 (MYF5)-positive somatic mesodermal progenitors [34,35]. In contrast, beige adipocytes are formed both through de novo adipogenesis and transdifferentiation of dormant white adipocytes in WAT in response to external and pharmacological stimuli such as chronic cold exposure and β-adrenalin receptor agonists [24]. A recent study revealed that beige adipocytes are de novo differentiated from fibroblastic progenitor cells with a smooth muscle cell (SMC) gene signature [24]. Single-cell analysis revealed that adipocyte progenitor cells (APCs) in WAT contain a proliferative subpopulation that contributes to beige adipocyte development [36]. The beige APCs uniquely express specific cell surface markers, such as CD81 and platelet-derived growth factor receptor alpha (PDGFRA), and SMC marker genes, such as *Acta2* and *Sm22*. Recent studies have clarified that two distinct types of beige adipocyte progenitors exist in perivascular regions: *Pdgfra*-positive fibroblastic progenitors and *Pdgfra*-negative *Myh11*-positive adipogenic SMCs [37]. A different group of researchers also identified that *Pdgfra*-negative and transient receptor potential vanilloid 1 (*Trpv1*)-positive vascular SMCs were progenitors that differentiated into thermogenic adipocytes in response to cold exposure [38].

**Figure 2:**
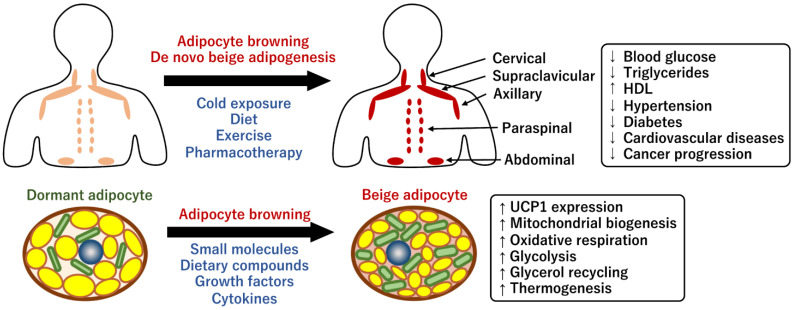
Anatomical distribution of human brown/beige adipocytes and their metabolic benefits. Human beige adipocytes are recruited through adipocyte browning of dormant adipocytes in specific WAT depots in response to cold exposure. The cold stimulus also likely promotes de novo beige adipogenesis from unique progenitor cells in these depots. Dietary compounds, exercise, and pharmacotherapy also contribute to these processes. Distinct metabolic benefits have been reported in individuals with detectable beige adipocytes. In in vitro cell culture systems, adipocyte browning is promoted by specific bioactive molecules including dietary compounds, growth factors, and cytokines; insights obtained from these studies could help uncover the molecular mechanisms underlying the direct regulatory action exerted by these molecules on adipocyte browning.

In human infants, the interscapular BAT (iBAT) plays a critical role in the prevention of hypothermia because the contribution of the shivering muscle is negligible due to less muscle mass [24]. The infant BAT gradually disappears during the progression of involution and is considered to be almost lost in adult humans in the long term. A recent article regarding single-cell analysis in rabbit iBAT reported the presence of the follistatin-like 1 (*Fstl1*) gene, which is a gene responsible for involution. This is intriguing because rabbits also undergo an involution progression similar to that in humans [39]. The classical brown adipose progenitor-specific loss of *Fstl1* gene in mice resulted in the partial loss of BAT and the incapacitation of the thermogenic functions, indicating that the expression of *Fstl1* is required for maintenance. In 2009, the technical development of 18F-fluorodeoxyglucose positron-emission tomography and computed tomography (18F-FDG PET/CT) identified metabolically active adipocytes around the neck and clavicle in adult humans [40,41,42]. BAT located around these regions may have evolved to protect the brain against hypothermia. The metabolically active adipocytes resembled mouse beige adipocytes rather than classical brown adipocytes in terms of gene expression and transient states of thermogenic capacity [43]. Human BAT is sporadically distributed in cervical, supraclavicular, paraspinal, and abdominal adipose depots [44] (Figure 2). The mass and activity of human BAT decline with age, which may be linked to reduced whole-body energy expenditure and fat accumulation [45]. In general, BAT is highly innervated by the SNS, which facilitates thermogenesis in response to adrenergic signaling via catecholamines such as norepinephrine and epinephrine [46]. Therefore, the proportion of brown adipocytes in the depots is correlated with the density of noradrenergic nerves [47]. As described earlier, the capacity of human beige adipocytes is initially determined by the number of beige progenitor cells in WAT [24]. However, it has remained ambiguous which mechanism, de novo biogenesis or adipocyte browning, contributes more to the formation of human beige adipocytes in the specific adipose depots [48]. Further studies are required to deduce methods to pharmacologically increase the potential browning capacity of adult humans.

In addition to glucose, fatty acids, and cellular triglycerides, BAT consumes other circulating substrates including succinate, branched-chain amino acids (BCAAs), and glutamate during thermogenesis, suggesting that BAT functions as a metabolic sink for clearance of circulating metabolites [24,49]. BAT selectively accumulates circulating succinate as an intermediate in the mitochondrial TCA cycle in response to cold exposure [50]. Oxidation by succinate dehydrogenase generates ROS, resulting in the activation of UCP1-mediated thermogenesis. Therefore, oral administration of succinate prevented high-fat diet (HFD)-induced obesity and glucose tolerance. Another report revealed that human BAT absorbed circulating BCAAs upon cold exposure and catabolized them in the mitochondria of brown adipocytes as fuel for the TCA cycle [51]. In addition, brown adipocyte-specific loss of BCAA catabolism promoted HFD-induced obesity and glucose intolerance. These results suggest that the clearance of BCAAs by BAT likely confers metabolic benefits because high levels of circulating BCAAs are associated with the pathogenesis of obesity and diabetes [52,53]. In addition, a study in which microdialysis catheters were inserted into human supraclavicular BAT revealed that cold exposure substantially increased the in vivo uptake of circulating glutamate [49,54]. Glutamate is then oxidized to α-ketoglutarate to enter the TCA cycle. However, glutamate uptake was lower than that of glucose during cold exposure, suggesting that glutamate is unlikely to be a major substrate for mitochondrial respiration. Therefore, an increase in glutamate uptake in human BAT contributes to anaplerosis.

### 2.3. Prevention of Human Metabolic Diseases through Brown Fats

An imbalance between caloric uptake and energy expenditure leads to overweight conditions and obesity [9]. Notably, mitochondria in brown fat coordinate systemic energy metabolism by actively consuming blood glucose and lipids, which is associated with the pathogenesis of obesity and related metabolic diseases including type 2 diabetes. Several studies have evaluated the physiological significance of BAT in energy expenditure, insulin sensitivity, body weight loss, white adipose tissue fibrosis, and hepatic steatosis [55,56,57]. Small prospective studies have indicated that short-term cold acclimation increases glucose uptake in human BAT and improves whole-body lipid metabolism in healthy and obese subjects [58,59,60,61,62,63], although applications of cold exposure are limited owing to adverse effects in patients with coronary artery disease [64]. Importantly, BAT exhibited a greater thermogenic capacity than abdominal WAT and uniquely induced the expression of lipid metabolic genes in response to cold exposure [62]. Other reports indicated that following cold exposure, human BAT improved systemic metabolic health by clearing circulating lipoproteins, acylcarnitines, and metabolites such as BCAAs [51,65,66]. A retrospective large cohort study clearly revealed that the presence of brown fat significantly reduced the risk of type 2 diabetes, dyslipidemia, coronary artery disease, cerebrovascular disease, cognitive heart failure, and hypertension [67]. In addition, the levels of blood glucose, triglycerides, and HDL in individuals with brown fat were significantly improved compared with those in individuals without detectable BAT. Notably, BAT was more beneficial to individuals with a higher body mass index (BMI). These observations suggest that human BAT exerts beneficial effects on systemic metabolic health independent of age, sex, and BMI. Furthermore, several studies in rodent models have reported that BAT transplantation enhances insulin sensitivity and decreases body fat mass in obese rodent models [68,69,70]; however, the underlying mechanism remains unclear. Generally, surgical procedures for BAT transplantation create new wounds and increase the risk of hematoma, seroma, and infection. Furthermore, since the accessibility of human BAT is limited due to its sparse distribution in specific adipose depots, identifying a sufficient source of autologous human BAT or thermogenic brown adipocytes is a hurdle for the clinical application of transplantation [71]. Taken together, ample evidence supports the therapeutic potential of BAT in whole-body metabolic homeostasis for preventing the widespread prevalence of obesity and its associated harmful effects.

Polycystic ovary syndrome (PCOS) is a common metabolic and endocrine disease that causes hyperandrogenism and metabolic and reproductive dysfunction [72]. Among women of reproductive age, approximately 5–20% are affected by PCOS, and 38–88% of women with PCOS have central adiposity or obesity, indicating that the pathogenesis of PCOS is closely related to obesity-related metabolic disorders [73]. Body weight loss by just 5% is known to cause a significant improvement in the reproductive and metabolic parameters of PCOS, implying that weight loss exerts therapeutic effects in obese women with PCOS [74]. Insulin-sensitizing agents such as metformin and medications such as SGLT2 inhibitors for the treatment of type 2 diabetes can be used to improve the metabolic aspects in patients with PCOS; however, their efficacy on weight loss is potentially limited [75]. This leaves BAT as the potential therapeutic target for the treatment of PCOS because the pathology and management of PCOS are closely related to the physiological roles of BAT [76]. BAT activity tends to decrease in patients with PCOS owing to high central adiposity and obesity [77]. A recent report indicated that cold exposure was beneficial for the treatment of PCOS as it reduced the levels of circulating testosterone and luteinizing hormone (LH), the expression of steroidogenic enzymes, inflammatory factors, and cystic ovarian follicles in rats with PCOS [78]. Additionally, BAT transplantation ameliorated PCOS phenotypes by improving insulin resistance and infertility in mice and rats [79,80]. However, both cold exposure and BAT transplantation are not yet available for most human patients with PCOS. Therefore, the activation of endogenous BAT and brown adipogenesis may become a therapeutic strategy for treating PCOS [81].

Obesity is associated with cancer development through its impact on inflammation, epithelial-to-mesenchymal transition, angiogenesis, and fibrosis [2]. The activation of BAT by cold exposure and β-adrenalin receptor agonist reduced circulating blood glucose levels, which resulted in improved survival of tumor-bearing mice due to reduced proliferation of cancer cells [82,83]. Moreover, the surgical removal of BAT or genetic ablation of UCP1 eliminated the suppressive effects on tumor growth. This evidence proves the significant impact of BAT on processes beyond thermogenesis and the corresponding metabolic benefits. A recent clinical study reported that breast cancer patients with BAT activity exhibited longer progression-free survival than those without BAT activity [84]. The manipulation of BAT activity may provide a therapeutic intervention to prevent not just obesity-related metabolic diseases but also cancer progression. Further studies are warranted to obtain more insights into the efficacy and methodology of reducing the progression of various types of cancers in human patients.

### 2.4. Human Brown Adipocyte Models

As described earlier, human brown/beige adipocytes are dispersed in the specific adipose depots; the distribution is highly varied among different individuals [67]. Hence, isolating a sufficient number of homogeneous beige adipocytes from different human specimens is substantially difficult [85]. In addition, beige adipocytes are transiently induced by external stimulation; hence, their activity and gene expression appear to be inconsistent under ex vivo cell culture conditions. Therefore, a cell model of human beige adipocytes is expected to provide more insights into the molecular mechanisms underlying adipocyte browning and the browning factors that selectively enhance the population in vivo. However, to date, only a few models of human brown adipocytes are established either from non-tumor specimens or cells that have not been genetically manipulated for immortalization [86,87]. In particular, human primary culture is required for a better understanding of normal metabolic pathways in beige adipocytes and to assess the physiological relevance between humans and mice. Although human beige adipocytes are not exactly the same as murine ones, most studies on the functions of beige adipocytes and their development have been performed using murine models.

In this context, our group developed chemical compound-induced brown adipocytes (ciBAs) derived from dermal fibroblasts as a novel model for human brown/beige adipocytes [88,89]. The direct conversion methodology is chemical-based and serum-free, which is ideal and convenient for performing reproducible experiments such as basic studies, drug screening, and clinical uses [90]. Transcriptional profiles for brown adipogenic reprogramming in ciBAs are closely associated with lipid metabolic and thermogenic functions of brown/beige adipocytes [91]. Transcriptome analysis comprehensively demonstrated that ciBAs underwent integrated changes in gene expression related to adipocyte browning, whereas fibrogenic gene expression in dermal fibroblasts was largely repressed. Human brown adipogenic gene signature in ciBAs is supported by the increased expression of adipocyte-enriched genes, including *UCP1*, *CIDEA*, *CITED1*, *MTUS1*, and *KCNK3* [43,92]. ciBAs exhibit enhanced mitochondrial levels and oxygen consumption rates (OCRs) in the mitochondria, which are the most important characteristics of brown adipocytes that enable the active consumption of fatty acids and glucose for thermogenesis [30]. Adipose tissue-derived mesenchymal stem cells (AdMSCs) are another option for a cell model for human primary cell culture that can differentiate into mature adipocytes [87]. Comparing adipocyte browning in AdMSC-derived adipocytes with that in ciBAs, almost the same sets of genes were either activated or repressed. However, AdMSC-derived adipocytes showed higher expression of UCP2 than in ciBAs, whereas ciBAs showed much higher expression of UCP1. Unlike UCP1, *UCP2* is ubiquitously expressed in most tissues such as the liver, heart, lung, brain, and pancreas [93,94], and it differentially contributes to the regulation of mitochondrial oxidative stress and energy metabolism [95,96]. This observation implied that ciBAs might have a phenotype closer to that of brown adipocytes than of the AdMSC-derived adipocytes.

Bioactive molecules targeting human brown/beige adipocytes could therapeutically enhance systemic energy metabolism by increasing their population and UCP1-mediated thermogenic capacity [97,98]. This is a promising strategy for the treatment of obesity and obesity-related metabolic diseases including diabetes mellitus and cardiovascular diseases. Since the availability of in vivo human brown adipocytes is limited, we performed a small-scale chemical screening using ciBAs and identified several compounds that enhance browning. One of these compounds is capsaicin, a well-known pungent alkaloid found in chili pepper [99]. Animal studies have reported that capsaicin or capsinoid supplementation improved glucose metabolism in both rodents and humans [100,101]. However, the anti-obesity effects of capsaicin undergo the indirect activation of the SNS through sensory neurons expressing TRPV1 [102]. Our reports demonstrated that treatment with capsaicin directly increased a series of browning effects in ciBAs [103]. Notably, the immortalized human brown adipocyte cell line, hTERT A41-BAT SVF [104] promoted browning following capsaicin treatment. In contrast, the AdMSC-derived adipocytes were not responsive to treatment with capsaicin in terms of UCP1 expression, indicating that the responsiveness to bioactive molecules and browning agents varied among the cell models. Thus, ciBA is a promising cell model for effective and reliable screening of human browning factors, especially, through TRPV1. A different report indicated that AdMSCs were differentiated into beige adipocytes on treatment with several growth factors and small molecules [105]. Drug discovery using the AdMSC-derived beige adipocytes identified several browning factors, indicating that AdMSCs are also useful for the identification of anti-obesity drugs and browning factors [99,105,106].

## 3. Thermogenic Regulation by Mitochondrial Dynamics in Brown Adipocytes

### 3.1. Thermogenic Regulation by UCP1

**Figure 3:**
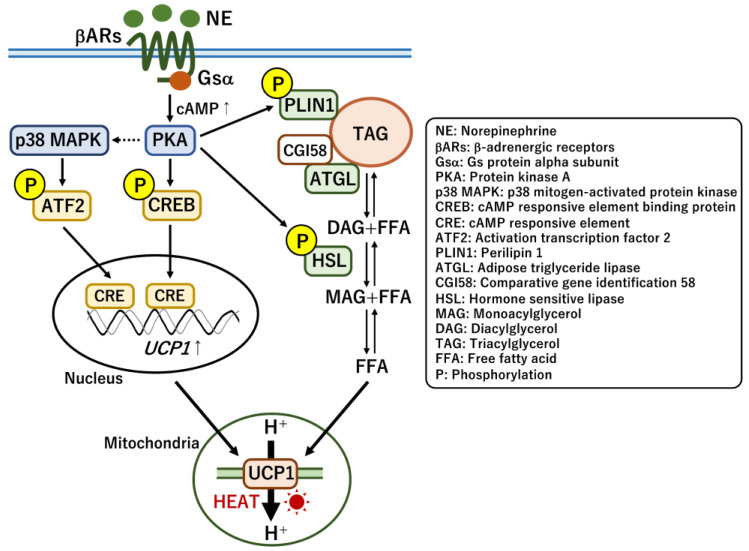
Regulatory mechanisms underlying UCP1-dependent thermogenesis in response to cold exposure. Upon cold exposure, norepinephrine (NE) is secreted from sympathetic nerve terminals. The activation of β-adrenergic receptors by NE increases the cellular levels of cAMP. The cAMP-dependent activation of protein kinase A (PKA) phosphorylates transcriptional factors, such as cAMP response element binding protein (CREB) and activating transcription factor 2 (ATF2), for the rapid activation of *UCP1* expression. PKA also phosphorylates hormone-sensitive lipase (HSL) and perilipin 1 (PLIN1) proteins for rapid lipolysis to supply mitochondria with free fatty acids (FFAs). The simultaneous induction of UCP1 expression and FFA supply enables acute heat production upon cold exposure.

UCP1-dependent thermogenesis plays a pivotal role in heat generation during cold exposure and the clearance of circulating metabolites [24]. Cold exposure stimulates the secretion of the catecholamine norepinephrine (NE) from SNS nerve terminals that are innervated within BAT. As a result of the response of β-adrenergic receptors to NE, adenylyl cyclase activity stimulated by the G protein Gsα increases cellular cAMP levels and drives an energy-burning thermogenic pathway for simultaneous enhancement of UCP1 expression, lipolysis, and mitochondrial biogenesis in brown adipocytes [107] (Figure 3). Protein kinase A (PKA) is activated by cAMP, and it phosphorylates cAMP response element binding protein (CREB) and activating transcription factor 2 (ATF2) via the activation of p38 mitogen-activated protein kinase (MAPK) for the subsequent transcription of thermogenic genes including UCP1 [108]. Phosphorylated CREB and ATF2 enable the acute transcription of *UCP1* by directly binding to the cAMP-responsive elements in the promoter region upon cold exposure [109]. In addition to CREB and ATF2, several nuclear receptors also control *UCP1* transcription in response to nutritional and hormonal conditions. Thyroid hormone receptors (TRs), peroxisome proliferator-activated receptors (PPARα and PPARγ), and retinoic acid receptors (RARs) directly regulate UCP1 expression by inducing the binding of their corresponding responsive elements to the promoter [110]. They are activated by endogenous ligands such as triiodothyronine (T3), unsaturated fatty acids, and vitamin A derivatives [111]. Moreover, retinoid x receptors (RXRs) form heterodimers with these nuclear receptors, which likely modulate their transcriptional activity. PPARα and PPARγ mainly control fatty acid oxidation and brown adipocyte differentiation, respectively, which also indirectly affect UCP1 expression. In addition, several key transcription factors including CCAAT/enhancer-binding proteins (C/EBPs), estrogen-related receptor α (ERRα), PR domain zinc-finger protein 16 (PRDM16), and zinc finger protein 516 (ZFP516) directly regulate *UCP1* transcription [112,113]. Thus, reverse genetic studies in mice have provided many insights into the molecular mechanism of *UCP1* expression; however, whether the same set of transcriptional factors regulates *UCP1* transcription in human brown adipocytes remains unclear. Some of these transcription factors recruit major epigenetic factors, such as histone methyltransferases MLL3/4 and EHMT1 and demethylase LSD1, for inducing histone modifications that regulate thermogenic genes including *UCP1* [110,114,115]. A recent study revealed that DNA demethylase TET1 potentially repressed thermogenic genes including *UCP1* and *PPARGC1A* in mouse beige adipocytes [116]. Mechanistically, the repression was largely DNA demethylase-independent, and TET1 modulated the activity of histone deacetylase HDAC1 on the promoter of *UCP1* and *PPARGC1A*. These observations suggest that *UCP1* transcription is strictly regulated by multiple transcriptional factors likely to coordinate the thermogenic capacity under different nutritional conditions.

In the thermogenic pathway, lipolysis is simultaneously activated by PKA-mediated phosphorylation of hormone-sensitive lipase (HSL) and perilipin 1 (PLIN1) proteins [117,118]. Phosphorylated PLIN1 releases comparative gene identification 58 (CGI58) and subsequently activates adipose triglyceride lipase (ATGL). The hydrolysis of triglycerides in lipid droplets was performed using ATGL and HSL as rate-limiting enzymes. However, BAT-specific deletion of either ATGL or the inhibitory regulator CGI58 suggested that the intracellular hydrolysis regulated by either of the proteins was not essential for thermogenesis [119,120]. Instead, the deletion of either protein in both adipose tissues (WAT and BAT) attenuated thermogenesis in BAT, indicating that the release of free fatty acids (FFAs) from WAT makes a more significant contribution to thermogenesis than does the de novo lipolysis in BAT. Thus, simultaneous induction of FFA release and UCP1 expression facilitates acute heat production to maintain body temperature upon cold exposure.

In addition to transcriptional regulation, recent studies have reported that UCP1 protein activity is regulated by post-translational modifications such as sulfenylation, succinylation, and malonylation [121]. A higher level of ROS generation occurs in response to cold exposure and adrenergic stimulation owing to electron leakage during mitochondrial respiration [122]. In this context, mitochondrial ROS alters the redox state of cysteine thiols in brown adipocytes and sulfenylated UCP1 protein at Cys253 [123]. Importantly, the pharmacological depletion of mitochondrial ROS and the mutation of Cys253 reduced norepinephrine-induced thermogenesis. In contrast, UCP1 activity and stability are negatively regulated via succinylation and malonylation by Sirt5, a mitochondrial desuccinylase and demalonylase [124]. Notably, these modifications are increased in BAT compared with those in WAT and are also increased by cold exposure and HFD feeding, suggesting that they might be involved in the regulation of UCP1 and mitochondrial protein turnover. Brown adipocyte-specific deletion of Sirt5 alters cold adaptation and fasting-induced mitophagy.

### 3.2. UCP1-Independent Non-Shivering Heat Generation

**Figure 4:**
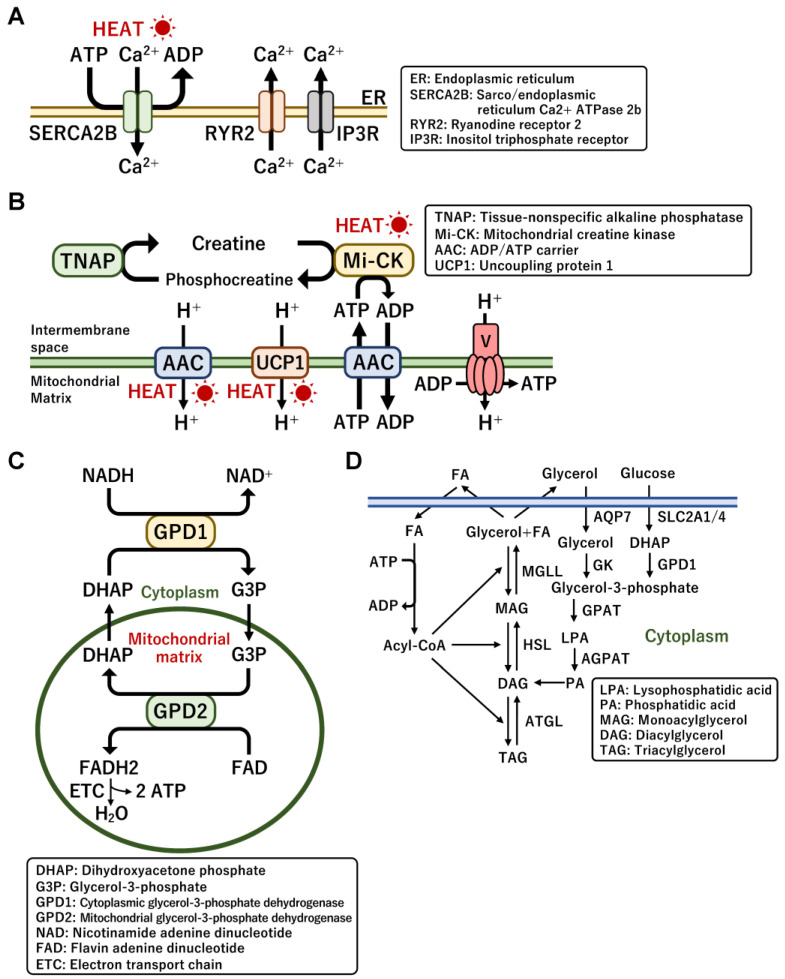
UCP1-independent and non-shivering thermogenic pathways. (**A**) The Ca^2+^ futile cycle is instigated by the sarco/endoplasmic reticulum Ca2^+^-ATPase 2b (SERCA2b) and ryanodine receptor 2 (RyR2) across the membrane of the endoplasmic reticulum (ER). (**B**) The creatine–phosphocreatine futile cycle is induced by the mitochondrial creatine kinase (Mi-CK) and tissue-non-specific alkaline phosphatase (TNAP) in mitochondria. ADP/ATP carrier (AAC) located in the mitochondrial inner membrane is a major transporter protein for the exchange of mitochondrial ATP and cytoplasmic ADP. Additionally, AAC also mediates mitochondrial uncoupling for thermogenesis. (**C**) The NADH-G3P shuttle is performed by two glycerol-3-phosphate (G3P) dehydrogenases located in the cytoplasm and mitochondria, allowing rapid ATP synthesis in mitochondria. (**D**) The lipolysis and re-esterification futile cycle is an ATP-consuming triglyceride synthesis pathway, which recycles glycerol and free fatty acids that are broken down from triglycerides. In all these metabolic reactions the intermediate metabolites are converted to the original substrate by consuming ATP, which leads to the release of energy as heat.

UCP1-deficient mice are known to be still tolerant to hypothermia, implying the presence of UCP1-independent non-shivering thermogenesis [26]. Major UCP1-independent mechanisms are mediated by the Ca^2+^ futile cycle across the endoplasmic reticulum (ER) and the creatine/phosphocreatine futile cycle in mitochondria, which is driven by the consumption of ATP produced by glycolysis and mitochondrial oxidation [117]. The Ca^2+^ cycling pathway substantially contributes to thermogenesis and glucose utilization during cold exposure, particularly in beige adipocytes generated within WAT but not in classical brown adipocytes [125,126]. The mechanism is driven by Ca^2+^ import and export between the cytoplasm and ER through the sarco/endoplasmic reticulum Ca2^+^-ATPase 2b (SERCA2b) and ryanodine receptor 2 (RyR2) (Figure 4A). Ca^2+^ is exported from the ER by RyR and inositol triphosphate receptor (IP3R) proteins, and Ca^2+^ is imported by SERCA2B in an ATP-dependent manner. ATP hydrolysis by SERCA2B results in energy dissipation and heat generation. Adipocyte-specific SERCA2-deficient mice lost heat production in WAT but not in iBAT [125]. Intriguingly, UCP1-independent thermogenesis appears to be evolutionarily conserved even in pigs that lack a functional UCP1 protein. In addition, the expression of SERCA2B was enhanced by cold exposure and norepinephrine or forskolin treatment. Cold-induced activation of α1-adrenergic receptor triggers Ca^2+^ release from the ER. These observations suggest that the Ca^2+^ cycle in beige adipocytes is involved in the control of thermogenesis.

The creatine futile cycling also functions in heat generation in mitochondria [127]. Proteome analysis of mitochondria isolated from classical BAT and inguinal WAT revealed that enzymes related to creatine metabolism were enriched specifically in WAT mitochondria. The creatine substrate is phosphorylated and dephosphorylated by creatine kinase (Mi-CK) and tissue-non-specific alkaline phosphatase (TNAP) in an ATP-dependent manner (Figure 4B). The fat-specific loss of glycine amidinotransferase (GATM), which is the rate-limiting enzyme for creatine biosynthesis, exhibited cold intolerance and low creatine levels and promoted diet-induced obesity due to the repression of energy expenditure that was induced in response to high-calorie feeding [128]. A recent report revealed that thermogenic fat adipocytes possessed TNAP with robust phosphocreatine phosphatase activity [129]. TNAP was uniquely localized in the mitochondria of brown adipocytes where the cycling occurs; however, the mitochondrial localization was not observed in non-thermogenic cell types such as hepatocytes. The expression and activity of TNAP were induced by cold exposure, indicating that the creatine cycle is physiologically involved in UCP1-independent thermogenesis in brown adipocytes. Importantly, adipocyte-specific loss of TNAP promoted HFD-induced body weight gain and reduced energy expenditure without a change in food intake [129]. A recent report suggested that the circadian control of creatine metabolism in brown fats was associated with diet-induced thermogenesis [130]. The increased creatine cycle prevented obesity caused by mistimed HFD feeding during the metabolically inactive period (light cycle) in the mice. However, the effects of creatine supplementation on cold-induced BAT activity and energy expenditure have not been detected in young adult vegetarians [131].

The mitochondrial ADP/ATP carrier (AAC) located in the inner mitochondrial membrane controls cellular ATP production by exchanging mitochondrial ATP for cytoplasmic ADP [132]. A recent report revealed that AAC mediated mitochondrial uncoupling, as does UCP1 [133] (Figure 4B). Importantly, AAC-mediated proton leakage was positively regulated by the presence of FFAs and negatively regulated by ADP/ATP exchange, suggesting a connection between mitochondrial energy dissipation and cellular ATP demand. Thus, AAC contributes to thermogenesis by regulating both mitochondrial uncoupling and coupling respiration. In addition, the nicotinamide adenine dinucleotide hydrate (NADH)-glucose-3-phosphate (G3P) shuttle has been proposed as a UCP1-independent thermogenesis pathway [134]. This allows mitochondria to rapidly obtain ATP in an aerobic manner, which is likely to support rapid heat production through both UCP1- and ATP-dependent heat production [135] (Figure 4C). G3P is reduced by cytoplasmic G3P dehydrogenase (GPD1) and oxidized by mitochondrial GPD (GPD2), leading to the transfer of protons to the mitochondrial respiration chain. However, the efficiency of ATP production (two ATP per oxygen) in the shuttle is worse than that in the regular ETC pathway (three ATP per oxygen), implying that the missing third ATP may be dissipated as heat [136]. Mice deficient in whole-body mitochondrial GPD2 had impaired energy expenditure and NE-induced thermogenesis in BAT, indicating the potential importance of the NADH-G3P shuttle [135].

The futile cycle of lipolysis and re-esterification is an ATP-dependent thermogenic pathway [134] (Figure 4D). Triglycerides are broken down into FFAs and glycerol. FFAs and G3P derived from secreted glycerol and the glycolysis pathway are re-esterified to triglycerides. The futile cycle functions as an ATP metabolic sink because the synthesis of fatty acyl-coenzyme A (CoA) consumes ATP during partial and full re-esterification [137]. In total, seven ATP molecules are consumed for every complete cycle, which leads to the release of heat [138]. Notably, substrate flux analysis indicated that the futile cycle was activated by adrenergic stimulation in primary mouse brown adipocytes, indicating that this cycle is involved in non-shivering thermogenesis [139]. The brown adipocyte-specific activation of glycerol kinase (GK) expression incorporated glycerol secreted due to lipolysis and reduced FFA secretion from adipocytes—also known as glycerol recycling [140]. Treatment with PPARγ agonists, such as thiazolidinediones and rosiglitazone, markedly activated the incorporation of secreted glycerol into triglycerides along with increased GK expression as one of the target metabolic genes [140]. All the metabolic reactions mentioned above eventually result in the conversion to the original substrate by consuming ATP and releasing energy as heat. These ATP-consuming futile cycles have a therapeutic potential to counteract obesity by modulating systemic energy homeostasis.

### 3.3. Mitochondrial Biogenesis and Dynamics in Brown Adipocytes

In brown fats, changes in mitochondrial number, shape, and motility regulate mitochondrial substrate oxidation for thermogenic capacity [20]. Increased mitochondrial biogenesis is observed during adipocyte browning and differentiation, which promotes thermogenesis and energy expenditure by accelerating the oxidation of FFAs as an energy source [141]. Cold exposure and subsequent NE secretion promote both the expression and activity of PPARγ coactivator 1α (PPARGC1A) in the role of a master regulator of mitochondrial biogenesis in brown adipocytes [142]. β-adrenergic signaling transcriptionally activates PPARGC1A and post-translationally activates it via p38 MAPK-mediated phosphorylation [143]. PPARGC1A binds to the LXXLL motif of nuclear receptors, including PPARγ and ERRα, and nuclear respiratory factors (NRFs) as a coactivator to transcriptionally activate metabolic genes involved in mitochondrial biogenesis and oxidative capacity [144]. PPARGC1A is also activated by adenosine monophosphate-activated protein kinase (AMPK)-mediated phosphorylation and nicotinamide adenine dinucleotide (NAD)-dependent deacetylase sirtuin-1 (SIRT1)-mediated deacetylation [145]. AMPK and SIRT1 activity is dependent on the AMP/ATP ratio and NAD level, respectively, which function as molecular links between mitochondrial biogenesis and cellular energy homeostasis [146,147]. The AMPK-PPARGC1A pathway regulates mitochondrial energy metabolism, which activates the expression of mitochondrial transcription factor A (TFAM) to increase mitochondrial DNA (mtDNA) transcription by upregulating NRF-1, NRF-2, and ERRα [142]. TFAM translocates PPARGC1A and NRFs into mitochondria and forms a complex with mtDNA [148]. Either overnutrition or obesity causes low AMPK activity owing to high levels of cellular ATP, which results in mitochondrial dysfunction [149]. The SIRT1-PPARGC1A pathway also upregulates the expression of TFAM in mtDNA replication [150]. Notably, the SIRT1-PPARGC1A pathway eliminates oxidative stress-mediated ROS production by activating mitochondrial antioxidants and mitophagy [151,152]. Furthermore, both PPARGC1A pathways are required for various energy regulating processes, such as exercise endurance, muscle-type fiber transformation, muscle atrophy, WAT browning, and thermogenesis.

Human brown adipocytes generally show fragmented morphology in mitochondria, which exerts higher catabolic processes, uncoupled respiration, and thermogenesis [153]. The combination of mitochondrial fission and induced UCP1 expression may enable more efficient thermogenesis in brown adipocytes. Several key proteins regulate mitochondrial fission and fusion. Dynamin-related protein-1 (DRP1) is a cytoplasmic GTPase, and the inhibition of its activity resulted in disrupted adrenalin-induced thermogenesis, thus demonstrating its potential role [153]. Norepinephrine secretion following cold exposure induced rapid mitochondrial fragmentation via PKA-dependent phosphorylation of DRP1, along with lipolysis and UCP1 induction [141]. In human MSC-derived beige adipocytes, DRP1-mediated fission contributed to increased uncoupling activity [153]. In contrast, the importance of mitochondrial fusion was confirmed in mice deficient in the mitochondrial fusion protein mitofusin 2 (MFN2) [154]. The brown fat-specific null mice showed lipohypertrophy in BAT and impaired cold-induced thermogenesis, suggesting that both mitochondrial fission and fusion play an important role in thermogenic capacity. Moreover, the brown fat-specific deletion of the inner mitochondrial membrane fusion protein optic atrophy 1 (OPA1) indicated that OPA1 caused mitochondrial dysfunction and impaired cold-induced thermogenesis [155]. A recent report revealed that OPA1-dependent fumarate accumulation promoted cell-autonomous adipocyte browning [156]. This evidence suggests that both mitochondrial biogenesis and dynamics substantially contribute to thermogenic activity in brown adipocytes.

### 3.4. Mitochondrial Turnover Regulated by Mitophagy in Brown Adipocytes

Mitophagy is a macroautophagic process that clears damaged mitochondria and contributes to thermogenic function in brown adipocytes [157]. Chronic cold stress stimulated ROS-mediated mitochondrial damage and mitophagy for mitochondrial turnover in BAT [158]. ATG5 is essential for autophagic vesicle formation, and BAT-specific ATG5 null mice could not maintain their body temperature during chronic cold stress. This indicates the essential role of mitophagy in adaptive thermogenesis and mitochondrial homeostasis in thermogenic fat. In addition, Beclin1 (ATG6) is required for nucleation of the phagophore, and adipose tissue-specific beclin1 knockout resulted in defective mitophagy, leading to dysfunctions in mitochondrial integrity, adrenalin-stimulated lipolysis, and thermogenic gene expression [159]. During the initiation of mitophagy, the ubiquitin-dependent PTEN-induced kinase 1 (PINK1)/Parkin pathway is activated in response to impaired mitochondrial membrane potential [22]. PINK1 phosphorylates Parkin (an E3 ubiquitin ligase) at Ser65 to recruit it to the outer membrane of the depolarized mitochondria. Intriguingly, Parkin-deficient mice displayed overactivated BAT and were protected against HFD-induced obesity [160,161]. Although Parkin expression was transcriptionally induced during brown adipocyte differentiation, thermogenic stimuli such as cold exposure and noradrenalin reciprocally repressed its expression in BAT [160]. Notably, mitophagy in mice reacclimated from cold to thermoneutral conditions was induced along with increased Parkin expression. This observation indicates a well-established mitochondrial quality control by Parkin to eliminate the mitochondria damaged by ROS following thermogenesis. Transcriptional repression of Parkin may be necessary to maintain sufficient mitochondria during thermogenesis in BAT. Mitophagy is also pathologically involved in the whitening process of beige adipocytes after the withdrawal of thermogenic stimuli [33]. Superfluous mitochondria that are no longer needed for thermogenesis are supposed to be eliminated by mitophagy. The genetic ablation of mitophagy in UCP1-expressing cells resulted in higher thermogenic capacity and protection against diet-induced obesity owing to the prevention of beige adipocyte loss [162]. Another report revealed that PKA-mediated phosphorylation of Parkin in response to β-adrenergic stimulation inhibited the recruitment to the outer membrane of mitochondria, which resulted in the maintenance of beige adipocytes during cold exposure.

In contrast, PINK1-mediated mitophagy is also required for the elimination of damaged mitochondria during cold exposure [163]. In addition, the use of UCP1-deficient mice revealed that cold-induced mitophagy in BAT was dependent on UCP1-mediated mitochondrial damage. In contrast, the brown adipocyte-specific deletion of PINK1 caused reduced energy expenditure and impaired thermogenesis due to the loss of damaged mitochondrial clearance [164]. Defective mitochondrial functions in the PINK1-deficient mice induced the NOD-like receptor family pyrin domain containing 3 (NLRP3) inflammasome, which failed to acquire a brown adipocyte-like phenotype in brown adipocyte precursors. The different phenotypes of Parkin- and PINK1-deficient mice indicate that a PINK1-independent mitophagy pathway is responsible for basal mitophagy that exists in highly metabolically active tissues including BAT [165]. A recent report also suggested that a set of serine/threonine protein kinases, STK3 and STK4, controlled adipocyte mitophagy in WAT rather than in BAT where the expression was repressed by cold exposure [166]. The genetic ablation of STK3 and STK4 increased thermogenic capacity and mitochondrial content due to reduced mitophagy in beige adipocytes. Pharmacological inhibition ameliorated obesity and improved metabolic profiles in mice. Therefore, these kinases are potential targets for the treatment of obesity-associated metabolic diseases, although their long-term inhibition may increase safety concerns.

## 4. Obesity and Mitochondrial Metabolism

### 4.1. Impact of Obesity on Human Brown Fat Activity

**Figure 5:**
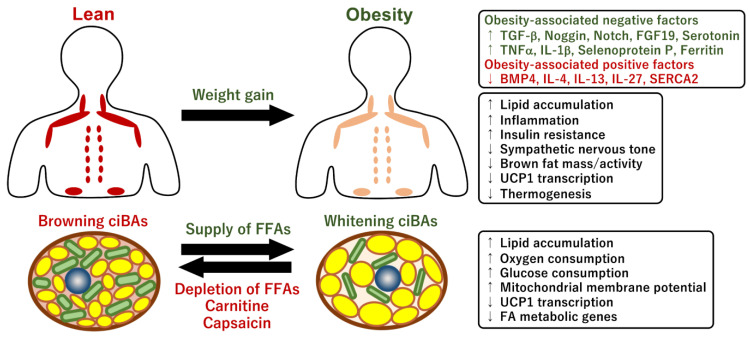
Obesity-associated metabolic differences in human individuals and the brown adipocyte model. Obese human individuals exhibit the repression of BAT mass and activity and lower BAT uptake of blood glucose and free fatty acids compared with those in lean individuals. Mechanistically, the repression is caused by obesity-associated factors that positively and negatively modulate adipocyte browning, de novo brown adipogenesis, UCP1 expression and activity, and adrenergic responses. The amount of the negative factors, such as TGF-β, Noggin, Notch, TNFα, and selenoprotein P, increases under obese conditions, whereas the amount of the positive factors such as BMP4 and Il-27 decreases. The pharmacological modulation of the metabolic pathways involved in these obesity-associated factors may provide therapeutic intervention in the management of obesity and metabolic diseases through brown fats. During the culture of the chemical compound-induced brown/beige adipocytes (ciBAs), the increase in free fatty acids (FFAs) in the culture medium induces white adipocyte-like phenotypes of ciBAs in terms of UCP1 expression and lipid metabolism, which may reflect BAT under obese conditions. In contrast, the depletion of FFAs or prolonged treatment with either carnitine or capsaicin causes the browning process of ciBAs, which may reflect BAT under lean conditions.

Obesity and overweight conditions dramatically affect systemic energy homeostasis by impacting lipid and glucose metabolism, insulin sensitivity, inflammation, and gut microbiota [167]. Typically, chronic inflammation, fibrosis, progenitor cell senescence, and catecholamine resistance are associated with hypertrophic adipocytes in patients with obesity [168]. Adipocyte browning is impaired by conditions associated with obesity and aging in both rodents and humans [169,170,171]. The accumulated evidence on the quantification of human BAT using 18F-FDG PET/CT scans has indicated that mass and activity are inversely related to BMI, visceral adiposity, and ages across cold and thermoneutral conditions [40,41,42,44,172,173] (Figure 5). Consistently, cold- and insulin-stimulated glucose uptake in human BAT was severely blunted in obese participants compared with that in lean participants [173]. In addition, the amount of cold-stimulated BAT was negatively correlated with the triglyceride content in BAT [174]. Fatty acid uptake was also higher in cold-stimulated BAT in lean rather than in obese participants [175]. Another report indicated that cold-induced thermogenesis, assessed by an increase in resting energy expenditure, was impaired in individuals who were overweight and obese [176]. Consistent with these observations, norepinephrine treatment did not promote beige differentiation of preadipocytes isolated from overweight adult patients [177]. UCP1 expression is known to be decreased in the WAT of obese mice [178,179,180]. The reduction in *UCP1* mRNA level was post-transcriptionally regulated through the process of mRNA decay by the deadenylase Cnot7 and its interacting partner Tob [178]. In human WAT, the expression levels of brown adipogenic genes were negatively correlated with BMI and fat mass and positively correlated with energy expenditure [181,182]. Furthermore, adipocytes differentiated from subcutaneous abdominal WAT derived from obese individuals had lower UCP1 protein levels than that from lean persons [183]. Another report indicated that brown adipogenic potentials, such as thermogenic gene expression, oxygen consumption rate, and lipolysis ability, were impaired in preadipocytes isolated from subcutaneous fat tissues of obese individuals [184]. Although gender differences in the prevalence of BAT are still controversial, the thermogenic response to cold exposure was greater in females than in males, implying that sex steroids might contribute to different thermogenic processes [185,186,187]. Altogether, this evidence suggests that lower mass and BAT activity in obese individuals may make them more intolerant to obesity and related metabolic disorders. A better understanding of the mechanisms underlying adipocyte browning and BAT activation in humans with obesity is required for the management of obesity.

### 4.2. Brown fat Activity Regulated by Obesity-Associated Factors

Adipocyte browning and mitochondrial metabolism in brown/beige adipocytes are affected by numerous obesity-associated hormonal and non-hormonal factors such as growth factors, inflammatory cytokines, circulating metabolites, metal ions, and neurotransmitters [188]. The activity or amount of these factors regulating adipocyte browning and development increased or decreased under obese conditions. The circulating levels of transforming growth factor-β (TGF-β) were increased in obese and overweight mice [189]. TGFβ signaling promotes the proliferation of preadipocytes, while TGFβ inhibits their differentiation by activating the Smad2/3 pathway. Accordingly, pharmacological and genetic ablation of TGF-β signaling resulted in resistance to diet-induced obesity and enhanced adipocyte browning [190,191,192]. In addition, the expression of TGF-β receptor 1 (Tgfbr1) and Smad3 was also increased in the WAT of HFD-induced obese mice, and adipocyte-specific deletion of Tgfbr1 promoted browning and protected against obesity, glucose intolerance, and hepatic steatosis [193]. However, the levels of bone morphogenic protein, BMP4, and antagonists such as Noggin and gremlin-1 decreased and increased, respectively, in the adipose tissue and plasma of obese human individuals [189,194,195]. As activated BMP signaling preferentially promotes adipogenesis through activation of the Smad1/5/8 pathway, higher levels of TGFβ and lower levels of BMP likely exacerbate adipose tissue dysfunction under obese conditions [189]. Thus, TGF-β and BMP signaling pathways regulate multiple aspects of white and brown adipocyte differentiation, adipose fibrosis, and lipid metabolism. An adipose tissue-specific Notch signaling null model also exhibited resistance to diet-induced obesity and elevated WAT browning, UCP1 expression, energy expenditure, and insulin sensitivity [196]. In addition, recent reports further suggested that pharmacological inhibition of Notch signaling by γ-secretase inhibitors promoted adipocyte browning and mitochondrial biogenesis and reduced subcutaneous fat tissue expansion [197,198,199]. Therefore, Notch signaling in thermogenic brown fats could be a therapeutic target in the management of diet-induced obesity and related metabolic diseases. The activation of the Notch signaling pathway by HFD in WAT and perivascular adipose tissue (PVAT) may be involved in obesity-associated repression of BAT [200,201].

The gut microbiota is closely involved in the management of obesity and metabolic conditions in humans [202]. Interestingly, the circulating level of FGF19, an enterokine secreted from ileal enterocytes, was negatively correlated with BMI and fat mass in obese human patients, whereas FGF19 levels were positively correlated with UCP1 expression in abdominal subcutaneous WAT [203,204]. Hepatic overexpression of FGF15 (FGF19 mouse ortholog) promoted adipocyte browning in inguinal WAT, whereas the loss of FGF15 inhibited it, suggesting that the enterokine FGF15/19 is an obesity-associated factor that regulates thermogenic brown fats. Another report showed that the amount of Firmicutes bacteria in the gut and plasma acetate levels were negatively correlated with UCP1 expression in subcutaneous fat in morbidly obese participants [205], indicating that gut microbiota might repress adipocyte browning via circulating acetate under obese conditions. In rodents, obesity was associated with an increased Firmicutes population in the gut [206].

In general, elevated inflammatory processes within WAT substantially reduce the capacity of brown fat differentiation and its metabolic functions [207]. Obesity-induced recruitment of inflammatory macrophages in WAT and the subsequent induction of pro-inflammatory cytokines are closely associated with the development of obesity [208]. In addition, some pro-inflammatory cytokines secreted by immune cells are involved in the regulation of thermogenic functions [209]. The infiltration of M1 macrophages in WAT inhibited cold-induced thermogenesis and UCP1 expression through the secretion of the pro-inflammatory cytokine tumor necrosis factor α (TNFα) [210]. The pharmacological elimination of M1 macrophages improved cold tolerance and UCP1 expression in obese mice, whereas injection of recombinant TNFα suppressed UCP1 expression. Another report indicated that activated macrophage-derived interleukin-1β (IL-1β) repressed cold- and adrenaline-induced UCP1 expression in WAT of obese mice [211]. A recent study showed that therapeutic administration of IL-27 protected against diet-induced obesity and glucose intolerance [212]. IL-27 directly activated UCP1 expression via the p38 MAPK-PGC1α signaling pathway in adipocytes. Notably, the circulating level of IL-27 was uniquely reduced within inflammatory cytokines in obese human subjects, suggesting that IL-27 is an obesity-associated factor for BAT activity. Consistent with these observations, a genome-wide association study indicated that single nucleotide polymorphisms in IL-27 were correlated with BMI and insulin resistance [213,214]. Furthermore, adipocyte browning was promoted by group 2 innate lymphoid cells (ILC2), which were recruited by IL-33 in human and murine WAT [215]. Mechanistically, the activation of ILC2 by IL-33 enhanced the proliferation of adipocyte progenitors and beige differentiation potential via the type 2 cytokines, IL-4 and IL-13 [216,217]. The population of ILC2 was decreased in both obese human participants and HFD-induced obese mice, implying that the reduction of ILC2 is involved in obesity-associated BAT dysfunction. In addition, serotonin, which is locally secreted from mast cells, inhibited browning in WAT [218]. Genetic and pharmacological inhibition of serotonin synthesis protected against obesity and elevated energy expenditure and adipocyte browning [219]. Since blood serotonin levels in obese human patients increased with the degree of obesity [220], serotonin might be involved in the regulation of BAT activity under obese conditions. These studies indicate that the functions of brown and beige adipocytes are negatively and positively regulated by several obesity-associated cytokines. Thus, inflammatory pathways are associated with the development of obesity, type 2 diabetes, heart disease, and cancer, possibly through the regulation of thermogenic brown fats.

### 4.3. Mitochondrial Metabolism Controlled by Metal Ions

The microenvironment surrounding brown adipocytes is closely associated with thermogenic functions. Metal ions critically contribute to the regulation of mitochondrial metabolism and thermogenesis. Selenium is involved in intracellular energy metabolism through its incorporation in the form of selenocysteine in several metabolic tissues including BAT [221]. In particular, a number of selenoproteins including the selenoprotein type 2 iodothyronine deiodinase (DIO2) are involved in the thermogenic regulation and mitochondrial energy metabolism. DIO2 is a key enzyme for the local conversion of thyroxine (T4) to its active form 3,5,5′-triiodothyronine (T3), which directly upregulates UCP1 expression during cold exposure [222]. A recent report on brown adipocyte-specific knockout of TRs indicated that T3 signaling was required for the thermogenic response of BAT for regulating gene expression involved in lipid and glucose metabolism [223]. Therefore, selenium deficiency may affect thermogenic functions in brown fats due to the reduced synthesis of thyroid hormones by DIO2 [224]. Besides UCP1 transcription, thyroid hormone signaling directly activates mitochondrial biogenesis, mitochondrial transport, the respiratory pathway, and the TCA cycle by upregulating the expression of PPARGC1A, which functions as a coactivator for TRs [223,225].

**Figure 6:**
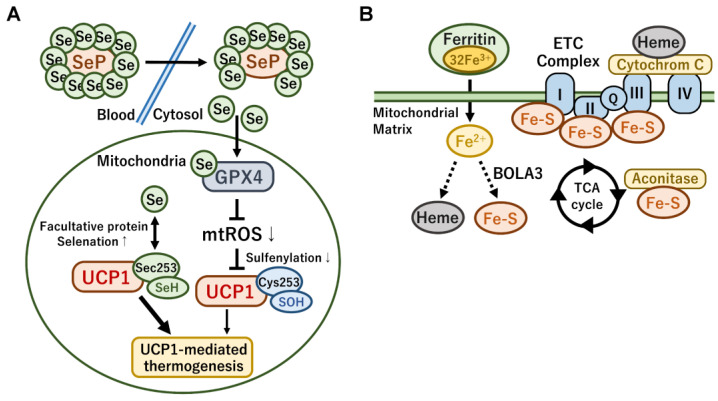
Selenium and iron are involved in mitochondrial energy metabolism and thermogenic capacity in thermogenic fat. (**A**) Circulating levels of selenoprotein P (SeP) are elevated under obese and diabetic conditions. Selenium molecules delivered by SeP into mitochondria activate glutathione peroxidase 4 (GPX4), which is a selenium-dependent antioxidant enzyme. The downregulation of mitochondrial ROS by GPX4 likely inhibits the activation of UCP1 through the sulfenylation at Cys253. In contrast, selenium is facultatively incorporated within the thiol residue of UCP1 at Cys253 to increase its activity. UCP1 with selenocysteine (Sec) is also sensitive to redox modification. Thus, selenium molecules multiply regulate UCP1 activity as well as thermogenic capacity in brown fat mitochondria. (**B**) Fe^2+^ is stored by the iron storage protein, ferritin, in an unstable Fe^3+^ form. In the mitochondrial matrix, Fe^2+^ is utilized for the formation of heme and iron–sulfur cluster (Fe-S) via the synthesis pathway including bola-like 3 (BOLA3). Heme is an essential component for cytochrome c in the electron transport chain (ETC), and the Fe-S cluster is required as a functional and structural component for the ETC complexes I, II, and III and aconitase in the tricarboxylic acid (TCA) cycle. Thus, iron plays a critical role in mitochondrial oxidative respiration and thermogenesis in brown fats.

Selenoprotein P (SeP) is a hepatokine responsible for the delivery of selenium into various cells and is an obesity-associated browning factor. The concentration of SeP was elevated in obese and diabetic conditions, which was associated with low BAT activity in humans [226]. The brown adipocyte-specific, but not liver-specific, deficiency of SeP enhanced cold resistance in mice. Mechanistically, SeP eliminated norepinephrine-stimulated mitochondrial ROS by transferring selenium to one of the glutathione peroxidases GPX4 (Figure 6A). The high levels of SeP repressed UCP1 activity by inhibiting sulfenylation at Cys235 via cold-induced mitochondrial ROS, suggesting that the management of circulating SeP levels and dietary selenium intake might be beneficial for the prevention of obesity through the modulation of UCP1-mediated thermogenesis in brown fats. In contrast, a recent report indicated that the sulfur in cysteine thiols was facultatively replaced with selenium [227]. Notably, dietary selenium supplementation elevated its incorporation at the Cys235 of UCP1 protein, which resulted in elevated BAT energy expenditure and resistance to HFD-induced obesity in mice. UCP1 protein with selenocysteine was sensitive to oxidative activation by ROS. Thus, selenium plays a pleiotropic role in the regulation of UCP1-mediated thermogenesis and mitochondrial biogenesis in brown fat.

Adaptive thermogenesis and whole-body energy metabolism are also regulated by iron-dependent pathways [228]. In particular, iron is required for the synthesis of heme and iron–sulfur clusters in the mitochondrial matrix (Figure 6B). Heme is a component of cytochrome c as part of the ETC, which is required for oxidative phosphorylation and energy expenditure in mitochondria [229]. The iron–sulfur cluster is an essential component of the ETC complexes I, II, and III and aconitase in the TCA cycle. Therefore, mitochondrial iron metabolism is essential for mitochondrial respiration and the thermogenic capacity of brown adipocytes. Ferritin is the major iron storage protein complex, and the serum levels of ferritin are used to estimate body iron storage level, which is associated with the development of insulin resistance and metabolic syndrome [230,231]. In agreement, a recent report indicated that mice fed with an HFD and an iron chelator deferasirox exhibited protective effects against diet-induced obesity along with elevated energy expenditure and beige adipogenesis in WAT [232]. Genetic and pharmacological inhibition of these iron metabolic pathways in mitochondria induced obesity along with decreased energy expenditure [233]. Brown fat-specific disruption of iron–sulfur cluster formation by bola-like 3 (Bola3) impaired norepinephrine-induced thermogenesis and whole-body energy expenditure. The study also revealed that iron–sulfur cluster formation was linked to an age-associated decline in energy expenditure through the mitochondrial lipoylation pathway. A recent report also implied that Bola3 was required for mitochondrial homeostasis and adrenaline-induced thermogenesis in both mouse beige adipocytes and human deep-neck brown fats [234]. In contrast, iron supplementation resulted in resistance to HFD-induced weight gain and hepatic lipid accumulation as the metabolic genes involved in the synthesis of heme and iron–sulfur clusters in the skeletal muscle and liver were upregulated [235]. Therefore, the manipulation of local and whole-body iron levels might be a therapeutic strategy for treating obesity and metabolic diseases in humans.

### 4.4. Mitochondrial Energy Metabolism and Transcriptional Regulation of UCP1

**Figure 7:**
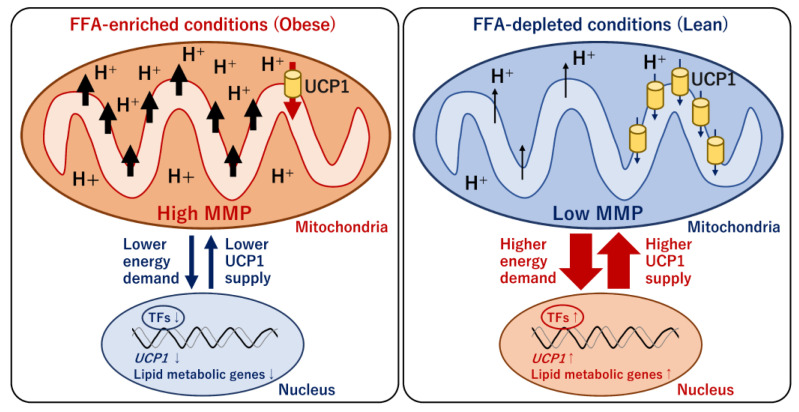
Feedback regulation between UCP1 gene expression and mitochondrial membrane potential (MMP) under different FFA conditions. FFA-enriched conditions induce MMP due to abundant fuels for mitochondrial oxidation; however, the expression of UCP1 and lipid metabolic genes is repressed (left panel). In contrast, FFA-depleted conditions result in lower MMP and the induced expression of UCP1 (right panel). Transcriptional factors such as CEBPA, PPARGC1B, RXRG, LXRA, and RORG exhibited a similar expression pattern to that of UCP1 and the metabolic genes between these FFA conditions; therefore, they may be involved in the feedback regulation. This feedback regulation may be required to coordinate UCP1-mediated thermogenesis under different mitochondrial energy statuses in brown adipocytes.

As mentioned earlier, the decreased mass and activity of BAT under obese conditions are mediated by multiple systemic factors whose expression, amounts, and activities differ between obese and lean conditions. These obesity-associated factors likely affect mitochondrial energy status and UCP1-mediated thermogenesis; however, the relationship remains unclear. Although circulating metabolites that increase under obese conditions are candidates for obesity-associated factors regulating brown adipocyte activity, all the components have been evaluated. Our recent report suggested that mitochondrial energy status determined the transcriptional level and mitochondrial content of UCP1 [236]. An increase in circulating FFAs is generally observed in obese individuals. To reflect obese and lean conditions in the cultures of a human brown/beige adipocyte model, ciBAs, bovine serum albumin with different levels of binding FFAs was added to the serum-free brown adipogenic medium [236]. The loss or lower levels of FFAs strongly activated *UCP1* transcription, whereas higher FFA levels substantially repressed transcription (Figure 7). The negative effect of FFAs on *UCP1* transcription was similar to that observed for previously tested FFA species such as oleic acid, palmitic acid, linoleic acid, α-linolenic acid, eicosapentaenoic acid, and docosahexaenoic acid. Under FFA-depleted conditions, cellular triglyceride accumulation and mitochondrial membrane potentials (MMPs) were reduced in ciBAs. Under these conditions, mitochondrial proton leakage was lower than that under normal FFA conditions, despite the much higher UCP1 content. This observation suggested that the induced expression of UCP1 might be required for the maintenance of consistent proton leaks under starvation or low-nutritional conditions. In contrast, high FFA levels repressed *UCP1* transcription along with elevated MMPs. UCP1 might be repressed to avoid excessive mitochondrial proton leakage under FFA-enriched conditions. Notably, the change in *UCP1* transcription was accompanied by changes in the expression of other metabolic genes involved in fatty acid and triglyceride biosynthesis pathways, such as GPAM, ACLY, SCD, SREBP1, and FASN. Thus, a unique feedback regulation of UCP1 expression counteracted the mitochondrial energy status altered by FFA availability in a human brown adipocyte model.

The detailed molecular mechanism underlying the relationship between mitochondrial energy status and *UCP1* transcription remains unclear. Similarly, prolonged treatment with carnitine activated the expression of UCP1 and lipid metabolic genes [236]. This was because carnitine accelerated mitochondrial FA oxidation, which caused the depletion of triglyceride storage in ciBAs. Interestingly, treatment with capsaicin also induced the expression of UCP1 and a similar set of lipid metabolic genes [103]. Prolonged capsaicin treatment resulted in lower MMP, implying that capsaicin exerted UCP1 expression by altering cellular lipid metabolism [236]. Our genome-wide transcriptional analysis suggested that the expression patterns of several transcription factors, such as *CEBPA*, *PPARGC1B*, *RXRG*, *LXRA*, and *RORG*, were similar to that of *UCP1*. Accumulating evidence suggests that these nuclear receptors are involved in the direct transcriptional regulation of *UCP1* [103,109]. Furthermore, a recent study reported that adipose tissue-specific knockout of PPARGC1B, a coactivator of nuclear receptors, impaired cold-induced thermogenesis [237]. The low expression of L-form optic atrophy (L-OPA1) in PPARGC1B-deficient mice reduced physical contact between mitochondria and lipid droplets in BAT, suggesting a significant role in mitochondrial energy metabolism. RXRG forms heterodimers with other nuclear receptors, such as PPARs, RARs, LXRs, and TRs, to elevate UCP1 expression [238]. CEBPA is also involved in *UCP1* transcription and adipocyte differentiation in concert with PPARγ [239,240]. Taken together, mitochondrial energy metabolism and *UCP1* transcription are closely connected to coordinate UCP1-mediated thermogenesis under different nutritional conditions. An on-target study is necessary to identify which transcription factors responsible for *UCP1* transcription are indirectly controlled by mitochondrial energy status. Further studies are warranted to demonstrate the biological significance of the feedback regulation in vivo.

## 5. Conclusions and Prospects

Emerging evidence has revealed the pronounced role of thermogenic brown fats in the treatment of obesity, cardiometabolic diseases, and cancers. Brown fat functions include enabling adaptation to hypothermia in mice and humans and acting as a metabolic sink for buffering excess nutrients and energy. Obesity is a worldwide problem, and combating it requires the urgent development of effective and sustainable therapies. Pharmacological activation of brown/beige adipocytes in the body is a promising strategy for the prevention of obesity. However, selective and effective anti-obesity drugs and dietary supplements targeting human brown adipocytes are still under development. To identify reliable compounds, the molecular mechanisms underlying human brown adipogenesis and adipocyte browning must be elucidated. As highlighted in this review, unique mitochondrial functions and energy metabolism in brown/beige adipocytes play central roles in thermogenesis and adipocyte browning. The mechanism underlying obesity-associated BAT repression in adult humans supports the physiological regulation of BAT activity. Further studies—both in vitro and in vivo—are required to identify selective browning factors and clarify their precise molecular mechanisms.

## Data Availability

No new data were created or analyzed in this study. Data sharing is not applicable to this article.
